# Dispersal-induced destabilization of metapopulations and oscillatory Turing patterns in ecological networks

**DOI:** 10.1038/srep03585

**Published:** 2014-01-07

**Authors:** Shigefumi Hata, Hiroya Nakao, Alexander S. Mikhailov

**Affiliations:** 1 Department of Physical Chemistry, Fritz Haber Institute of the Max Planck Society, Faradayweg 4-6, 14195 Berlin, Germany; 2 Department of Mechanical and Environmental Informatics, Tokyo Institute of Technology, Ookayama 2-12-1, 152-8552 Tokyo, Japan

## Abstract

As shown by Alan Turing in 1952, differential diffusion may destabilize uniform distributions of reacting species and lead to emergence of patterns. While stationary Turing patterns are broadly known, the oscillatory instability, leading to traveling waves in continuous media and sometimes called the wave bifurcation, remains less investigated. Here, we extend the original analysis by Turing to networks and apply it to ecological metapopulations with dispersal connections between habitats. Remarkably, the oscillatory Turing instability does not lead to wave patterns in networks, but to spontaneous development of heterogeneous oscillations and possible extinction of species. We find such oscillatory instabilities for all possible food webs with three predator or prey species, under various assumptions about the mobility of individual species and nonlinear interactions between them. Hence, the oscillatory Turing instability should be generic and must play a fundamental role in metapopulation dynamics, providing a common mechanism for dispersal-induced destabilization of ecosystems.

A rich variety of nonequilibrium pattern formation is supported by reaction-diffusion processes. One of the universal mechanisms of such pattern formation is provided by the Turing instability^1^; a diffusion-induced instability of the homogenous state which leads to spontaneous development of self-organized patterns. The Turing instability can play an important role in biological morphogenesis and has been extensively studied in various applications, including biological^2^,^3^,^4^,^5^,^6^, chemical^7^,^8^ and physical systems^9^. The classical Turing instability leads to the establishment of stationary spatial patterns. However, the oscillatory analogue of this instability is possible and it has also been discovered by Turing^1^. This oscillatory intability produces traveling or standing waves and therefore it is often called “the wave instability”^10^. At least three species are needed for the oscillatory instability, while the stationary instability is possible already with two species. The stationary Turing instability has been extensively studied both theoretically^2^,^3^,^4^,^9^ and experimentally^5^,^6^,^7^,^8^, whereas the oscillatory instability is more rare and it was found only for special chemical systems^11^,^12^,^13^. Note that complex spatio-temporal patterns can also emerge as a result of interactions between the stationary Turing instability and other bifurcations. For example, near the Turing-Hopf bifurcation point, complex mixed modes leading to standing waves and spatio-temporal chaos can exist^14^. However, their mechanism is different from that of the oscillatory Turing instability.

Reaction-diffusion processes are also characteristic to ecological systems. The reactions correspond in this case to the predator-prey and other interactions between the species. Both passive diffusion and active random migration are possible in ecological populations. Moreover, there are situations when a population occupies a large habitat and therefore can be considered as an extended spatial system. The classical Turing instability is possible in ecosystems. It has been shown that such instability should be generic for the two-species predator-prey models^15^ (see also^16^). Complex spatio-temporal ecological dynamics related to the Turing-Hopf^15^,^17^ and Turing-Takens-Bogdanov^15^ bifurcations has been discussed. Stationary Turing patterns have been found in realistic models describing plant-parasite^18^, plankton-fish^19^ and plant-insect^20^ interactions. The oscillatory Turing instability is also possible in ecology. In a study of a three-species plant-parasite-hyperparasite system, such instability leading to standing waves, has been previously considered^18^.

While some ecological systems can be described by reaction-diffusion equations for continuous media, there are also many ecosystems that are spatially fragmented and represent networks^21^,^22^,^23^. Such networks are formed by individual habitats which are linked by dispersal connections. Ecological species populate the habitats and diffusively migrate over a network. Such network-organized ecosystems are known as metapopulations^24^,^25^,^26^. The metapopulation concept has been applied to describe and investigate real ecological systems (see, e.g.^27^,^28^,^29^). It has also been used in the context of the epidemic research^30^,^31^,^32^,^33^. In the framework of the metapopulation concept, the role of dispersal connections in enhancing the stability of an ecosystem (*the rescue effect*) has been discussed^25^ (see also^28^). The theoretical results have been tested in the experiments with specially prepared metapopulations^27^,^29^.

Ecological metapopulations provide examples of reaction-diffusion systems with a network structure. Such systems can however be also found in other research fields. For instance, a biological embryo can be viewed as a network of cells with the chemicals diffusing over the pattern of intercellular connections^34^,^35^,^36^. Networks formed by coupled reactors can also be considered^37^,^38^. Theoretical studies of reaction-diffusion processes on networks have already attracted much attention^39^. Effects of infection spreading over the networks have been discussed in detail^30^,^31^,^32^,^33^. The role of network topology on the phase diagrams of nonequilibrium phase transitions on networks has been considered^40^. Traveling and pinned fronts in networks of diffusively coupled bistable elements were analyzed^41^ and control of front propagation by global feedback has been considered^42^. There is large literature on the networks formed by diffusively coupled oscillators^43^,^44^ (see also^45^). The role of dispersal connections in the synchronization effects in ecological networks has also been discussed^46^.

The stationary Turing instability for networks has been first analyzed in 1971 by Othmer and Scriven^34^. The authors have introduced a general mathematical description of the classical Turing instability in two-component reaction-diffusion networks and have applied their theory for regular lattices^35^. Stationary Turing patterns in small networks of coupled chemical reactors have subsequently been discussed^37^. The properties of such instability and of the final established stationary patterns in large random networks of diffusively coupled activator-inhibitor elements have been investigated and the mean-field theory of Turing patterns in such network systems has been constructed^9^. The global feedback control of the stationary Turing patterns in networks has been studied^47^. A detailed mathematical analysis of the hysteresis phenomena related to the network Turing bifurcation has recently been performed^48^.

In this article, we present, for the first time, theoretical investigations of the oscillatory Turing instability (the analogue of the wave bifurcation) in network-organized reaction-diffusion systems and apply them to large ecological networks. Our numerical simulations are performed for the metapopulations with various three-species food webs under different assumptions about the nonlinearities of the population dynamics. The instability could be found for all such systems and, as we therefore believe, it should be common in ecology. In contrast to the wave instability in continuous media, traveling or standing waves do not develop and oscillations, localized on a subset of network nodes, are instead observed. The bifurcation is supercritical and therefore the final pattern is usually well described by the first critical mode. This diffusion-induced instability leads to destabilization of metapopulations and the extinction of some species may result from it.

We consider ecological networks formed by individual populations which occupy separate habitats, labeled by indices 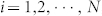, and are coupled by dispersal connections. Our attention is focused on the populations which consist of three interacting species. All possible food webs with three different species are displayed in Fig. 1. Generally, such ecological networks are described by equations
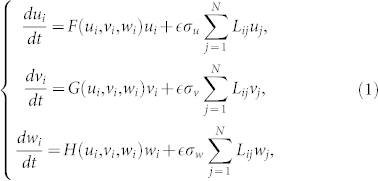
for 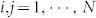, where population densities of species on node *i* are denoted as *u_i_* = [*U*]*_i_*, *v_i_* = [*V*]*_i_*, and *w_i_* = [*W*]*_i_*, functions *F* = *Q^u^* − *R^u^*, *G* = *Q^v^* − *R^v^*, *H* = *Q^w^* − *R^w^* are the differences of reproduction (*Q*) and death (*R*) rates for each species, and *σ_u_*_,*v*,*w*_ are the mobilities of the three species; the common parameter 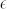 is introduced for convenience, so that the mobility of all species can be varied without changing relative mobilities. The Laplacian matrix **L** has elements 
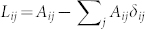
 where *A_ij_* is the matrix of connections between the habitats. We assume that, in absence of diffusive coupling, a stable stationary state (*u*_0_, *v*_0_, *w*_0_) exists which is determined by *F*(*u*_0_, *v*_0_, *w*_0_) = *G*(*u*_0_, *v*_0_, *w*_0_) = *H*(*u*_0_, *v*_0_, *w*_0_) = 0 where *u*_0_ > 0, *v*_0_>0 and *w*_0_>0.

For comparison, the continuous analog of the model (1) will be also considered, where node variables *u_i_*, *v_i_* and *w_i_* are replaced by space-dependent densities *u*, *v* and *w*. The diffusion processes are described by using the diffusion operator ∇^2^ instead of the Laplaciam matrix *L_ij_*. Thus, the model equations are given by
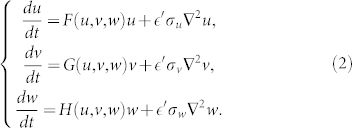
We examine three different food webs shown in Fig. 1a–c. In the food chain shown in Fig. 1a, top predator *W* is feeding on intermediate species *V* which is in turn a predator for prey *U*. In the food web shown in Fig. 1b, both species *V* and *W* play a role of the predators for prey *U* while *V* is also a prey for *W*. In the food web shown in Fig. 1c, species *U* and *V* are the prey for predator *W*. Explicit expressions for the reproduction rates and death rates in each model (A, B and C) are provided in Methods section. While different nonlinear dependence can be considered for predator-prey interactions, we will employ the Holling type II functions^3^,^49^ (See Supporting Information for other dependences). Note that the food web shown in Fig. 1d is excluded from our analysis. In such system, two competing species *V* and *W* cannot coexist in a steady uniform state and oscillatory Turing instabilities are impossible.

## Results

### Linear stability analysis

Below we give the precise definition of the oscillatory Turing instability in ecological networks. The stability of the uniform state of the model can be analyzed by the linear stability analysis. Small perturbations (*δu_i_*, *δv_i_*, *δw_i_*) are introduced to the steady state as (*u_i_*, *v_i_*, *w_i_*) = (*u*_0_, *v*_0_, *w*_0_) + (*δu_i_*, *δv_i_*, *δw_i_*). Substi tuting this into Eqs. (1), the following linearized differential equations are obtained:
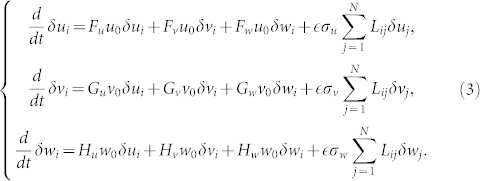
where 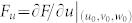, 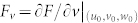, 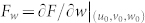, … are partial derivatives at the uniform steady state (*u*_0_, *v*_0_, *w*_0_). The Laplacian eigenvectors {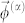} are introduced to decompose the perturbations. They are defined as
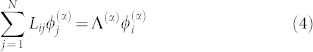
where Λ^(*α*)^ is the Laplacian eigenvalue of the *α* th mode (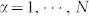). The mode indices {*α*} are sorted in the increasing order of the Laplacian eigenvalues {Λ^(*α*)^} so that 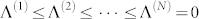 holds. The perturbations (*δu_i_*, *δv_i_*, *δw_i_*) are expanded over the set of the Laplacian eigenvectors as
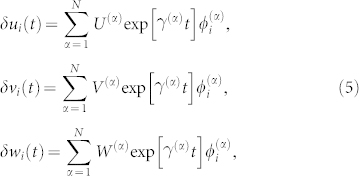
where 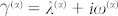 is a complex growth (or decay) rate of the *α* th eigenmode. Substituting these expressions into Eqs. (3), the following equations are obtained for each eigenmode:
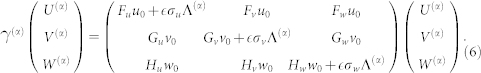
Thus, the decay rate of each eigenmode is determined by the characteristic equation
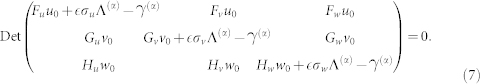
The uniform steady state is stable if *λ*^(*α*)^<0 for all *α*. The Turing instability takes place if *λ*^(*α*)^ becomes positive at some *α* = *α_c_* which represents the critical mode for the instability. The critical modes are stationary, 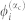, if 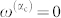. On the other hand, the critical modes can also be oscillatory, 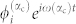, if 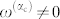. As noticed already by Turing^1^, oscillatory instabilities are possible only if the number of species is at least three.

For the continuous analogue (2), a similar analysis can be performed. Since eigenvectors of the differential operator ∇^2^ are plane waves, ∇^2^*e^ikx^* = −*k*^2^*e^ikx^*, small perturbations can be decomposed over them as
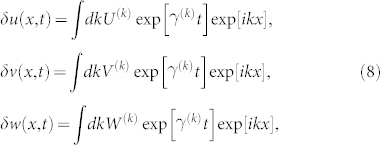
to obtain the characteristic equation 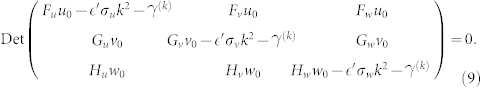
The instability occurs if *λ*^(*k*)^ becomes positive at some critical wave number *k*_c_. The critical mode corresponds to a traveling wave 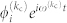 if 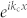 or to a stationary wave 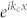 if 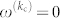.

For continuous media, the oscillatory Turing instability is usually called the wave instability, since the first unstable modes are the traveling waves. In networks, traveling waves do not appear and therefore it is not appropriate to talk about a wave instability in this case.

### Numerical investigations

First, we show the results for Model A. Starting from equal mobilities *σ_u_* = *σ_v_* = *σ_w_*, we gradually increase the mobility *σ_u_* of the bottom prey *U* and observe the oscillatory Turing instability. The stationary steady state becomes unstable above a certain threshold and non-uniform oscillations develop. The emerging oscillatory Turing pattern is displayed in Fig. 2a. One can notice that oscillations in the pattern are localized on a subset of nodes. Network nodes with relatively high degrees (hubs) exhibit oscillations while the other nodes remain staying near the uniform steady state (See also Supplementary videos S1 and S2). We will show later that such localized oscillations are typical for the oscillatory Turing patterns in networks. Remarkably, the stationary Turing instability can also be found in the same model when the mobility *σ_v_* of the intermediate species *V* is decreased (Fig. 2b).

Numerical simulations of the continuous model yield the behavior shown in Figs. 2c,d, where periodic boundary conditions are employed. The stationary pattern (Fig. 2b) becomes transformed to a periodic stationary Turing pattern (Fig. 2d), whereas the localized oscillatory pattern (Fig. 2a) gives rise to a traveling wave (Fig. 2c). While patterns in networks and continuous media may appear different, they indeed correspond to the same, stationary or oscillatory, Turing bifurcations.

**Figure 1:**
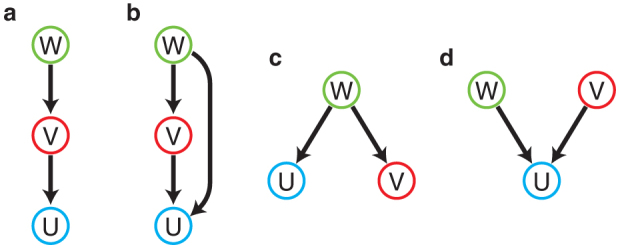
Food web diagrams. Each arrow goes from the consuming to the consumed species. In the last food web (d), no persistence with fixed values of 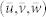 can be achieved; therefore this web is excluded from our analysis.

The oscillatory Turing instability is observed also in other food webs, Models B (Eqs. (12)) and C (Eqs. (13)). As well as in Model A, the instability takes place in each system when the mobility of one species is increased to exceed a certain threshold. In Model B, the oscillatory Turing instability occurs when the mobility *σ_u_* of the bottom prey *U* is increased. The emerging pattern is shown in the left panel of Fig. 3a. In Model C, the instability occurs as the mobility *σ_v_* of a prey *V* is increased up (Left panel in Fig. 3b). Thus, the oscillatory Turing instability is observed in all possible food webs with three species shown in Fig. 1a–c.

Furthermore, in our numerical simulations, the oscillatory Turing instablity was not sensitive to the choice of nonlinear functions in Eqs. (1). As shown in Supplementary Information, different nonlinear functions could be used to describe predator-prey interactions. We could observe the oscillatory Turing instability under various assumptions^3^,^49^ about the functional form of the reproduction and death rates. Moreover, the instability could be observed even if one of the three species was immobile (see Supplementary Information).

Therefore, we found the oscillatory Turing instability in a wide range of ecological models and conclude that the oscillatory Turing instability is *generic* for ecosystems. This discovery agrees with the results of our analytical investigation on the sufficient conditions for the instability (See Ref.^50^ and Supplementary Information). Examining emergent patterns, one can notice that developing oscillations in all considered systems are localized on a subset of network nodes with close degrees. Although the localizing nodes are different depending on a system, localized oscillations were always found. As we discuss below, localization is a common characteristic of oscillatory Turing patterns in ecological networks.

### Subcritical vs. supercritical bifurcations

Above the instability threshold, nonlinear effects become important. They can lead to the saturation of growth and the establishment of a final pattern. Generally, they also determine whether a bifurcation is subcritical or supercritical. When the bifurcation is subcritical, the pattern with large magnitude becomes immediately established once the instability threshold is exceeded. Such bifurcations are characterized by hysteresis, so that the pattern persists even below the instability threshold. In contrast to this, a supercritical bifurcation does not show a hysteresis and the magnitudes of established patterns are small close to the threshold. In this case, the final patterns do not differ much from the first critical modes near the bifurcation point.

Figure 4 displays the results of the linear stability analysis and the global amplitude 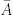 (See Methods) for the oscillatory (Fig. 4a,b) and stationary (Fig. 4c,d) Turing instabilities in Model A. Increasing the mobility *σ_u_* of the prey *U*, we calculated the linear growth rate 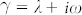 to find the instability at a threshold *σ_u_*_,crit._. The critical eigenmode has a complex linear growth rate (Fig. 4a) and therefore the instability is oscillatory. After passing the instability, we gradually increase the mobility *σ_u_* further away from the threshold and calculate the global amplitude 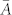 (Fig. 4b). As can be clearly seen in the figure, the oscillatory Turing instability corresponds to a supercritical bifurcation. We have checked that near the threshold 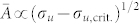 holds. Thus, small amplitude patterns can be established near the instability threshold. In contrast, the stationary Turing instability, which corresponds to a growth rate of a real number (Fig. 4c), exhibits a subcritical bifurcation. As shown in Fig. 4d, once the instability takes place, the amplitude 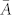 jumps up to a large value.

### Estimations of oscillatory Turing patterns using critical Laplacian eigenvectors

Due to its supercritical bifurcation, oscillatory Turing patterns near the instability threshold are well described only by the first critical eigenmode *α*_c_, so that we have
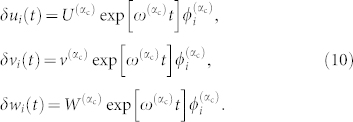
Therefore, in this case, final patterns can be predicted by means of the critical Laplacian eigenvectors 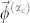.

In Fig. 5, we demonstrate the prediction for Model A. Figure 5a shows the results of the linear stability analysis at two different values of overall mobility 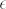 in Model A. The respective critical Laplacian eigenvectors are displayed in Figs. 5c,d and the actual oscillatory Turing patterns in Figs. 5e,f. As seen in Fig. 5, critical Laplacian eigenvectors and developing oscillatory patterns are localized on subsets of network nodes with close degrees. The localization for the considered network is demonstrated in Fig. 5b, where magnitudes |
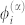
| of the components of Laplacian eigenvectors 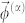 are displayed as a function of *α*. This localization is consistent with the previous discovery for large random networks^9^,^51^. The critical eigenmode *α*_c_ depend on the overall mobility 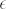 shown in Fig. 5a. As discussed previously^9^, the Laplacian eigenvalue Λ^(*α*)^ appears in the characteristic function (7) multiplied by 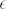. Then, if we change the overall diffusion mobility 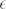, the critical eigenmode *α*_c_ shifts so that the product 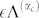 is kept constant. Thus, the characteristic localization of the critical Laplacian eigenvector changes depending on the overall mobility 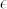 shown in Fig. 5c,d. Correspondingly, in emerging oscillatory Turing patterns, localizing nodes shift from hubs (Fig. 5e) to peripheral nodes (Fig. 5f). Thus, examining Fig. 5, one can predict the emerging patterns by means of respective critical Laplacian eigenvectors.

The oscillatory Turing instabilities found in other models are also identified with supercritical bifurcations shown in Fig. 3, and Figs. S1 and S2 in Supplementary Information. Thus, the instability generically leads to pattern formations of small amplitudes, which are described by the Laplacian eigenvectors of critical modes. This indicates that the localization property of the oscillatory patterns originates in the localizing Laplacian eigenvectors.

**Figure 2:**
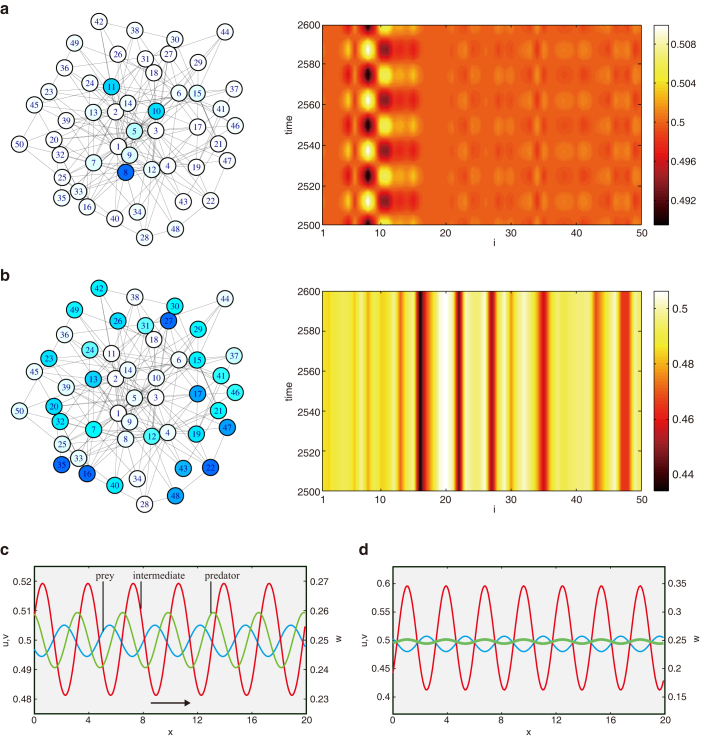
Examples of oscillatory (a,c) and stationary (b,d) Turing patterns in networks (a,b) and in the respective continuous media with periodic boundary conditions (c,d). In panels (a) and (b), mean oscillation amplitudes for all network nodes (see Methods, higher amplitudes are indicated by the deeper blue color) and time evolution diagrams are displayed. For continuous media, instantaneous concentration profiles are shown (c,d). The parameters are (a) *σ_u_* = 0.535, *σ_v_* = *σ_w_* = 0.01, 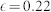, (b) *σ_u_* = *σ_w_* = 1, *σ_v_* = 0.022, 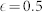, (c) *σ_u_* = 0.535, *σ_v_* = *σ_w_* = 0.01, 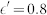, (d) *σ_u_* = *σ_w_* = 1, *σ_v_* = 0.022, 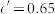.

**Figure 3:**
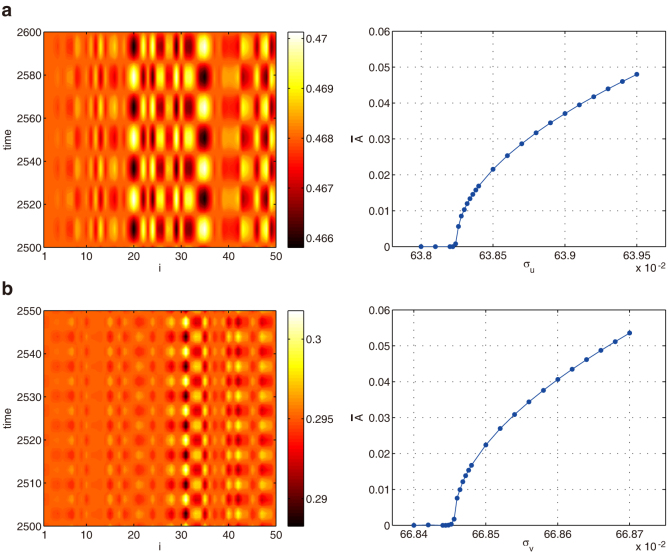
Oscillatory Turing instabilities in Model B and C. Left panels show final oscillatory Turing patterns. Right panels show the global amplitude 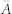 as a function of *σ_u_*. Dispersal mobilities are (a) *σ_v_* = *σ_w_* = 0.01 and 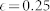. (b) *σ_v_* = *σ_w_* = 0.01 and 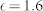. For the final patterns, (a) *σ_u_* = 0.6384 and (b) *σ_u_* = 0.6685 are used.

**Figure 4:**
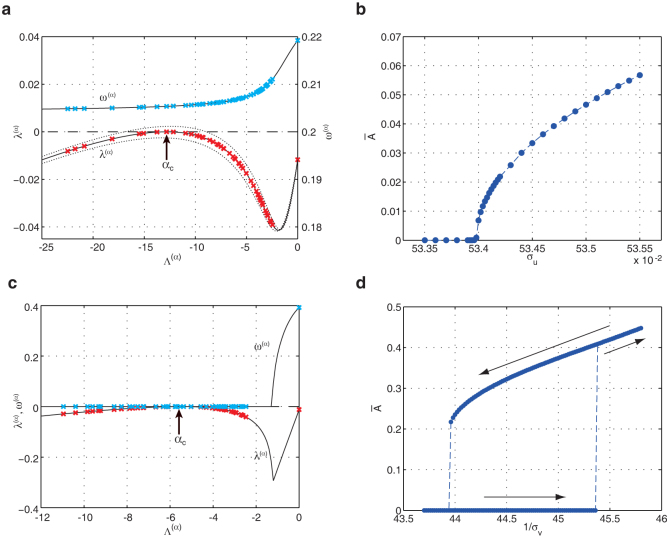
Linear stability analysis on Model A. Growth rates *λ*^(*α*)^ and frequencies *ω*^(*α*)^ for different modes *α* near the instability threshold (a,c) and the global amplitudes 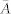 (b,d) for oscillatory (a,b) and stationary (c,d) Turing instabilities. The arrows in (d) show the directions of the parameter change. Dispersal mobilities are (a) 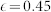, *σ_u_* = 0.5342, *σ_v_* = *σ_w_* = 0.01, (b) 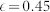, *σ_v_* = *σ_w_* = 0.01, (c) 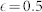, *σ_v_* = 0.022, *σ_u_* = *σ_w_* = 1, (d) 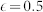, *σ_u_* = *σ_w_* = 1.

**Figure 5:**
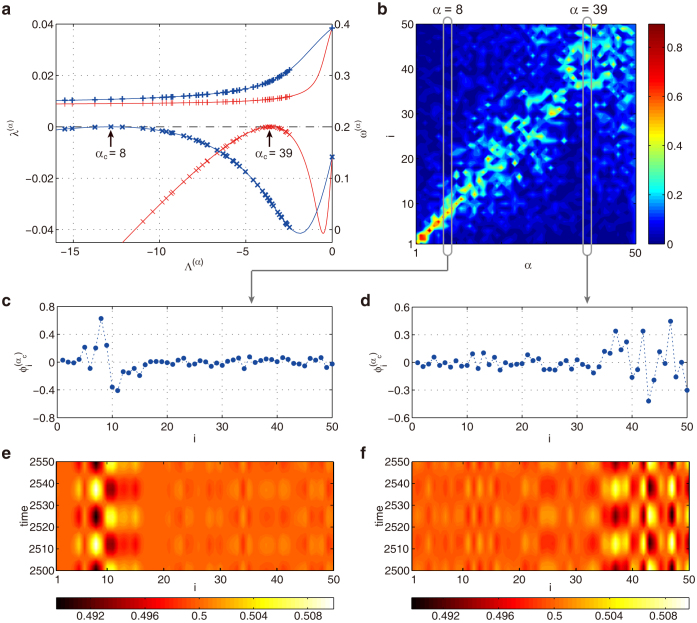
Oscillatory Turing instability in Model A. (a) Dependences of *λ*^(*α*)^ and *ω*^(*α*)^ on the Laplacial eigenvalue Λ^(*α*)^ for different overall mobilities, 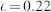 (red curves) and 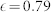 (blue curves); relative mobilities are *σ_u_* = 0.535, *σ_v_* = *σ_w_* = 0.01 for both cases. (b) Laplacian spectrum of the network is graphically displayed. Each column corresponds to one eigenvector *ϕ*^(*α*)^. Nodes are sorted according to their degrees *k* and the magnitude 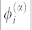 for each node *i* is indicated by using a color code. The mode indices {*α*} are sorted in the increasing order of the Laplacian eigenvalues {Λ^(*α*)^} so that 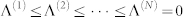 holds. (c–f) Critical Laplacian eigenvectors with (c) *α* = 8 and (d) *α* = 39 and the respective final oscillatory Turing patterns (e,f).

In contrast to the oscillatory instability, the stationary Turing instability corresponds to a subcritical bifurcation. This gives a striking difference between patterns arising from both the instabilities. It has been previously found in two-component reaction-diffusion networks that the stationary Turing instability corresponds to a subcritical bifurcation^9^. This holds also in three-component systems, that is, ecological networks with three species. The bifurcation is subcritical and the stable nonlinear state is far from the uniform steady state. Although differentiation starts from the nodes with the characteristic degree of the critical eigenvector, it takes place in surrounding nodes sequentially and spreads over the entire network. The amplitude of the developed final pattern becomes large. Thus, as we have seen in Fig. 2, although both instabilities are induced by the diffusion effect, the uniform steady state is destabilized in distinctly different fashions.

### Secondary instability - Extinction of ecological species

**Figure 6:**
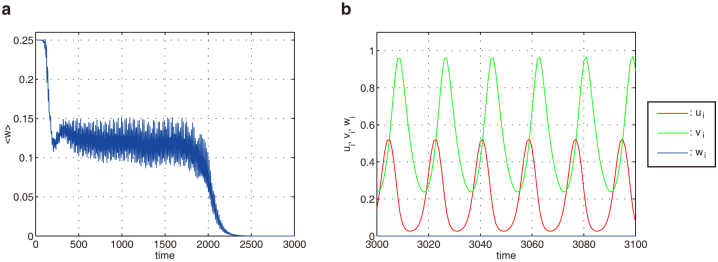
Secondary instability in Model A. (a) Time-dependence of the network average 〈*ω*(*t*)〉 far from the instability threshold. (b) Local densities of three species after a transient. The same dynamics are observed in all network nodes (Uniform oscillations). Dispersal mobilities are 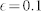, *σ_u_* = 6.75, *σ_v_* = *σ_w_* = 0.01.

We have studied the dynamical behavior induced by the oscillatory Turing instability far from the threshold and found a potential secondary instability, leading to the extinction of ecological species. Note that the dynamical behavior far from the instability threshold largely depends on the considered model. We have identified such extinction in Models A and B. As an illustration, we here show the results for Model A. Figure 6 displays numerical results far from the first instability threshold. Now, all eigenmodes are unstable except for the zero eigenmode *α* = 0 (Hopf mode). In Fig. 6a, the network average 〈*w*(*t*)〉 for the top predator *W* is plotted as a function of time. After an oscillatory transient, the average 〈*w*(*t*)〉 tends to zero and therefore the top predator vanishes (See also Supplementary videos S3). Two remaining species, the prey *U* and the intermediate predator *V*, exhibit uniform oscillations after the extinction of the top predator *W* (Fig. 6b). Thus, the secondary instability may be inherent in the oscillatory Turing instability, which destabilizes the considered ecosystem and leads to the extinction of ecological species.

## Discussion and conclusions

The mathematical description for the classical (stationary) Turing instability in networks has been proposed already in 1971^34^. However, it has been first applied only to regular lattices^34^,^35^ and small networks^37^,^38^. Recently, such instability in large random networks has been investigated and characteristic properties of stationary Turing patterns in large networks have been discussed^9^,^47^,^48^. In contrast to this, the mathematical theory of the oscillatory Turing bifurcation (the analogue of the wave bifurcation) in networks has been missing and our work is the first report where such theory is constructed.

Similar to continuous reaction-diffusion systems, the oscillatory instability needs three reacting species^1^ and it occurs when diffusion mobilities of the species are largely different. However, wave patterns, which are typical for such instability in continuous media, do not emerge in networks. Therefore, we prefer not to use the term “wave instability” in the present study. As we find, heterogeneous oscillations spontaneously develop and they are localized on a subset of network nodes with similar degrees. The localization of developing oscillations could be explained by taking into account the known statistical properties of Laplacian eigenvectors in large random networks^9^,^51^. Such eigenvectors correspond to the critical modes of the oscillatory Turing instability and hence they determine the properties of developing patterns.

Previously, it has been shown that the classical (stationary) Turing bifurcation in networks is subcritical; it is characterized by strong hysteresis and the properties of final stationary patterns in networks are largely different from those of the first critical modes. In contrast to this, we have found that the oscillatory Turing bifurcation in networks is typically supercritical. Thus, the hysteresis is absent and the amplitude of the Turing patterns gradually grows as the control parameter is increased. Near the bifurcation point, oscillatory Turing patterns with small amplitudes are observed and they agree well with the first critical modes. This means that by considering Laplacian eigenvectors the properties of final small-amplitude patterns can be analyzed.

While the proposed mathematical theory is general and applicable to systems with various origins, our detailed numerical simulations have been performed for the models of ecological networks also known as metapopulations^26^. We have considered metapopulations with the graph structure of scale-free networks. The habitats, representing network nodes, were occupied by local three-species ecosystems forming food webs. All possible food webs with three predator or prey species were considered. We have only excluded the food web with two predators and one prey shown in Fig. 1d because steady coexistence of all three species is not possible in this case. Moreover, simulations under various assumptions for nonlinear predator-prey interactions have been carried out.

The oscillatory Turing instability could be observed for all considered metapopulation models. It has been found as the network mobility (dispersal rate) of one of the species was gradually increased. Note that it could be the mobility of a prey or a predator, depending on the food web and the mathematical model applied. The instability has been observed also in the three-species metapopulations where one of the species was immobile. The results of our numerical simulations are supported by the general sufficient conditions for the oscillatory bifurcation in three-component ecological systems which we have derived. While our analysis has been performed only for systems with three species, it can be straightforwardly extended to the systems with a larger number of components and hence for the metapopulations with more complex food webs. Moreover, such instabilities are also possible in other biological and chemical systems. As two examples, numerical simulations for the chemical extended Brusselator and Oregonator models are presented in Supplementary Information.

We conclude that the oscillatory Turing instability may be *generic* for ecosystems. It should be generally expected whenever metapopulations with at least three species and sufficiently large differences in the mobilities of the species are investigated. This result may be of principal importance. Previously, the discussion has been focused on the role of dispersal connections in enhancing the stability of uniform steady states^25^,^27^. We find however that dispersal connections would often destabilize the uniform steady state and lead to the development of oscillations on a subset of network nodes. We would like to stress that such oscillations are a consequence of the differential dispersal mobilities of species and they were always absent for isolated populations in individual habitats.

While the developing oscillations are weak near the instability threshold, their amplitude increases with the distance from the critical point and large-amplitude oscillations are also possible. We have shown that the secondary instabilities of such oscillations may take place and, in the considered example Fig. 6, they have resulted in the extinction of one of the species and, therefore, in the degradation of an ecosystem. So far, only the global extinction through the development of uniform oscillations via a Hopf bifurcation has been discussed. Our work provides a different scenario for the extinction of species through the development of dispersal-induced hegerogeneous oscillations in ecological networks.

It would be interesting to perform experimental or field studies of the oscillatory Turing instability and the resulting Turing patterns in real ecological systems. For this purpose, it may be beneficial to work first with the artificially constructed metapopulations as it has been done before in the experimental studies on the role of dispersal connections^27^,^29^,^52^. To prove the presence of oscillatory Turing patterns in natural ecosystems, the development of such patterns and their responses to local perturbations should be analyzed, similar to what has been done to demostrate the existence of classical stationary Turing patterns in biological organisms^6^.

## Methods

### Food-web models

#### zwModel A

In the food chain shown in Fig. 1a, top predator *W* is feeding on intermediate species *V* which is in turn a predator for prey *U*. The collective dynamics of such metapopulation is described by Eqs. (1) with
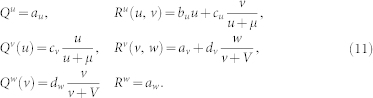
The parameters in Eq. (11) are fixed at *a_u_* = 1, *b_u_* = 1, *c_u_* = 1, *a_v_* = 1/4, *c_v_* = 1, *d_v_* = 1, *a_w_* = 1/2, *d_w_* = 1 and *μ* = *ν* = 1/2, yielding a uniform stationary state (*u*_0_, *v*_0_, *w*_0_) = (1/2, 1/2, 1/4).

#### Model B

In the food web shown in Fig. 1b, both species *V* and *W* play a role of the predators for prey *U* while *V* is also a prey for *W*. Such system is modelled as follows:
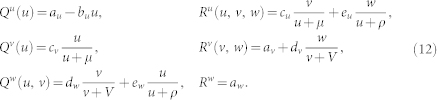
We fixed parameters in Eqs. (12) at *a_u_* = 1, *b_u_* = 1.5, *c_u_* = 1, *e_u_* = 0.4, *a_v_* = 0.25, *c_v_* = 1, *d_v_* = 1, *a_w_* = 0.5, *d_w_* = 1, *e_w_* = 0.4 and *μ* = *ν* = *ρ* = 0.5, which gives a uniform steady state 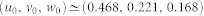.

#### Model C

In the food web shown in Fig. 1c, species *U* and *V* are the prey for predator *W*. Thus we have
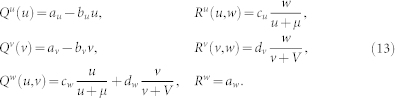
Parameters in Eqs. (13) are fixed at *a_u_* = *a_v_* = 5.55, *b_u_* = *b_v_* = 1.2, *c_u_* = *d_v_* = 3, *a_w_* = 1, *c_w_* = 1.5, *d_w_* = 1.5 and *μ* = *ν* = 2 in Eqs. (13), yielding a uniform steady state 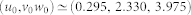.

### Network architecture

The network structure is determined by a symmetric adjacency matrix **A**, whose elements *A_ij_* are 1, if a link from node *j* to node *i* exists, and 0 otherwise. The degree, number of links, of node *i* is defined as 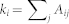. The network-Laplacian matrix **L** is defined as *L_ij_* = *A_ij_* − *δ_ij_k_j_*. The scale-free network is generated by the Barabási-Albert preferential attachment algorithm^53^. The network size was *N* = 50 and the mean degree was 〈*k*〉 = 8. For convenience, the node index {*i*} is sorted as decreasing order of the degree *k_i_*, number of links, so that 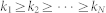 holds.

### Amplitude

To quantify emergent patterns, the amplitude is introduced. The amplitude of individual node *i* at time *t* is defined as
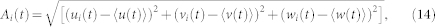
where 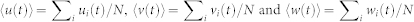 are the mean quantities. The long-time-averaged amplitude is defined as
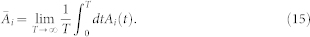
Similary, the amplitude of the entire network at time *t* is defined as
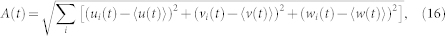
The long-time-averaged amplitude is defined as
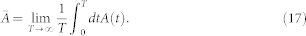
Setup of numerical simulations

The 4th-order Runge-Kutta scheme with time step Δ*t* = 10^−4^ was employed in numerical integration. The integration code was written by using the C language. The simulations were started from the uniform steady state (*u*_0_, *v*_0_, *w*_0_) with random small perturbations with standard deviations (*u*_0_, *v*_0_, *w*_0_) × 10^−3^. The steady state was identified by using the *Mathematica* software.

## Author Contributions

S.H., H.N. and A.S.M. designed the study, carried out the analysis and contributed to writing the paper. S.H. performed numerical simulations.

## Supplementary Material

Supplementary Information Supplementary Information

Supplementary Information Supplementary Move 1

Supplementary Information Supplementary Move 2

Supplementary Information Supplementary Move 3
