# Supplementary material for: Dispersal-induced destabilization of metapopulations and oscillatory Turing patterns in ecological networks
Source: Sci Rep. 2014 Jan 7;4:3585. doi: 10.1038/srep03585 (PMC3882759; doi:10.1038/srep03585)
Supplement: Supplementary Information [file srep03585-s1.pdf]

Dispersal-induced destabilization of metapopulations and oscillatory Turing patterns in ecological networks
# - Supplementary Information -

Shigefumi Hata,[1] Hiroya Nakao,[2] and Alexander S. Mikhailov[1]

[1]*Department of Physical Chemistry, Fritz Haber Institute of the Max Planck Society, Faradayweg 4-6, 14195 Berlin, Germany.*
[2]*Department of Mechanical and Environmental Informatics,*
Tokyo Institute of Technology, Ookayama 2-12-1, 152-8552 Tokyo, Japan

Contents:

I. Sufficient conditions for the oscillatory Turing instability in ecological networks

II. Numerical simulations of ecological networks

III. Numerical simulations of chemical reaction-diffusion networks

Supplementary video files showing oscillatory Turing patterns in ecological networks are available.

I. SUFFICIENT CONDITIONS FOR THE OSCILLATORY TURING INSTABILITY IN ECOLOGICAL NETWORKS

In our recent work [1], we considered general continuous reaction-diffusion systems with three species and derived sufficient conditions for the oscillatory Turing instability in continuous media. The derivation was based on the linear stability analysis. This analysis can be straightforwardly extended to the case of discrete networks. Below, we only reformulate the previously derived sufficient conditions for the networks and apply them to three-species ecological models;

$$\begin{cases} \dfrac{du_i}{dt} = F\left(u_i, v_i, w_i\right) u_i + \epsilon\sigma_u \displaystyle\sum_{j=1}^{N} L_{ij} u_j, \\ \dfrac{dv_i}{dt} = G\left(u_i, v_i, w_i\right) v_i + \epsilon\sigma_v \displaystyle\sum_{j=1}^{N} L_{ij} v_j, \\ \dfrac{dw_i}{dt} = H\left(u_i, v_i, w_i\right) w_i + \epsilon\sigma_w \displaystyle\sum_{j=1}^{N} L_{ij} w_j. \end{cases} \tag{S1}$$

Suppose that the mobilities σ_u and σ_v of the species U and V are fixed (and at least one of them is non-vanishing) and we gradually increase the mobility σ_w of species W. It follows from our previous analysis [1] that the oscillatory Turing instability will always be found at sufficiently high mobility σ_w if the following three sufficient conditions are satisfied:

$$F_u u_0 + G_v v_0 > 0, \tag{S2a}$$

$$F_w H_u u_0 + G_w H_v v_0 < 0, \tag{S2b}$$

$$\mathrm{Det}\begin{pmatrix} F_u u_0 + \sigma_u \Lambda & F_v u_0 \\ G_u v_0 & G_v v_0 + \sigma_v \Lambda \end{pmatrix} \neq 0 \text{ for any } \Lambda < 0, \tag{S2c}$$

where $(u, v, w) = (u_0, v_0, w_0)$ is a uniform stationary state determined by $F(u_0, v_0, w_0) = G(u_0, v_0, w_0) = H(u_0, v_0, w_0) = 0$, and $F_u = \partial F/\partial u|_{(u_0,v_0,w_0)}$, $F_v = \partial F/\partial v|_{(u_0,v_0,w_0)}$, $F_w = \partial F/\partial w|_{(u_0,v_0,w_0)}$, ... are partial derivatives at the uniform steady state. Note that one of the species U or V may be immobile, i.e. either $\sigma_u = 0$ or $\sigma_v = 0$.

If the mobilities σ_v and σ_w of the species V and W are fixed (and at least one of them is non-vanishing) and we gradually increase the mobility σ_u of species U, the oscillatory Turing instability will always be found at sufficiently high mobility σ_u if the following three sufficient conditions are satisfied:

$$G_v v_0 + H_w w_0 > 0, \tag{S3a}$$

$$G_u F_v v_0 + H_u F_w w_0 < 0, \tag{S3b}$$

$$\mathrm{Det}\begin{pmatrix} G_v v_0 + \sigma_v \Lambda & G_w v_0 \\ H_v w_0 & H_w w_0 + \sigma_w \Lambda \end{pmatrix} \neq 0 \text{ for any } \Lambda < 0. \tag{S3c}$$

Here, one of the species V or W may be immobile so that either $\sigma_v = 0$ or $\sigma_w = 0$.

When the mobilities σ_w and σ_u of the species W and U are instead fixed (and at least one of them is non-vanishing) and we gradually increase the mobility σ_v of species V, the oscillatory Turing instability will always be found at sufficiently high mobility σ_v if the following three sufficient conditions are satisfied:

$$H_w w_0 + F_u u_0 > 0, \tag{S4a}$$

$$H_v G_w w_0 + F_v G_u u_0 < 0, \tag{S4b}$$

$$\mathrm{Det}\begin{pmatrix} H_w w_0 + \sigma_w \Lambda & H_u w_0 \\ F_w u_0 & F_u u_0 + \sigma_u \Lambda \end{pmatrix} \neq 0 \text{ for any } \Lambda < 0. \tag{S4c}$$

Here, one of the species W or U may be immobile so that either $\sigma_w = 0$ or $\sigma_u = 0$.

Thus, if conditions (S2), (S3) or (S4) are satisfied, the oscillatory Turing instability will be found in the respective ecological networks as the mobilities σ_u, σ_v or σ_w is increased. Taking Model A as an example, Jacobian matrix at the steady state is

$$J = \begin{pmatrix} -1/4 & -1/2 & 0 \\ 1/4 & 1/8 & -1/2 \\ 0 & 1/8 & 0 \end{pmatrix}, \tag{S5}$$

which satisfies the sufficient conditions (S4) irrespective of the mobilities σ_v and σ_w of the two species V and W. Then, starting from equal mobilities $\sigma_u = \sigma_v = \sigma_w$, we gradually increase the mobility σ_u of the bottom prey U and find the oscillatory Turing instability as shown in the main text.

Note that the prey death rates R should be increasing functions of the predator densities. Hence, in the food chain (Fig. 1a in the main text), we have $\partial R^u/\partial v > 0$ and $\partial R^v/\partial w > 0$ and therefore $F_v = \partial F/\partial v < 0$ and $G_w = \partial G/\partial w < 0$. Moreover, the predator reproduction rates should generally increase with the prey densities. This implies that $\partial Q^v/\partial u > 0$ and $\partial Q^w/\partial v > 0$ so that we have $G_u = \partial G/\partial u > 0$ and $H_v = \partial H/\partial v > 0$. Thus, the following inequalities are satisfied.

$$F_v < 0,\ F_w = 0,\ G_w < 0,\ G_u > 0,\ H_w = 0,\ H_v > 0. \tag{S6}$$

In the food web shown in Fig. 1b in the main text, both species V and W play a role of the predators for prey U while V is also a prey for W. Therefore, $Q^u = Q^u(u), R^u = R^u(u, v, w), Q^v = Q^v(u, v), R^v = R^v(v, w), Q^w = Q^w(u, v, w)$ and $R^w = R^w(w)$. Hence, we have $\partial R^u/\partial v > 0, \partial R^u/\partial w > 0, \partial R^v/\partial w > 0, \partial Q^v/\partial u > 0, \partial Q^w/\partial u > 0$ and $\partial Q^w/\partial v > 0$. This leads to the conditions

$$F_v < 0,\ F_w < 0,\ G_w < 0,\ G_u > 0,\ H_u > 0,\ H_v > 0. \tag{S7}$$

In the food web shown in Fig. 1c in the main text, species U and V are the prey for predator W. Now, we have $Q^u = Q^u(u), R^u = R^u(u, w), Q^v = Q^v(v), R^v = R^v(v, w), Q^w = Q^w(u, v, w)$ and $R^w = R^w(w)$. Therefore $\partial R^u/\partial w > 0, \partial R^v/\partial w > 0, \partial Q^w/\partial u > 0$ and $\partial Q^w/\partial v > 0$. This implies

$$F_v = 0,\ F_w < 0,\ G_w < 0,\ G_u = 0,\ H_u > 0,\ H_v > 0. \tag{S8}$$

Thus, in all considered food webs, the inequalities

$$F_v G_u \le 0, \quad G_w H_v \le 0 \quad \text{and} \quad H_u F_w \le 0 \tag{S9}$$

hold and at least one of the inequalities holds strictly. We notice that they imply that the conditions (S2b), (S3b), and (S4b) are all satisfied. Therefore, we can conclude that, in contrast to general reaction-diffusion models, at least one of the three sufficient conditions will be *always satisfied* for ecological networks with three species. Hence, the oscillatory Turing instability should be more common in ecology, as compared with chemical systems.

II. NUMERICAL SIMULATIONS OF OTHER ECOLOGICAL MODELS

Here we show the results for different ecological models. Different dependence of reproduction and death rates on the densities of the species in predator-prey models are possible. In the main text, Holling type II dependences have been used as a typical example. Below we give models with other dependences including Holling type III [2, 3] and the functions introduced by Murray [3]. As the food web architecture, the food chain shown in Fig. 1a of the main text is employed.

Model D. The food chain (Fig. 1a) with the Holling type III dependence for both prey and predator:

$$\begin{aligned} Q^u(u) &= a_u - b_u u, & R^u(u,v) &= c_u \frac{uv}{u^2+\mu}, \\ Q^v(u) &= c_v \frac{u^2}{u^2+\mu}, & R^v(v,w) &= a_v + d_v \frac{vw}{v^2+\nu}, \\ Q^w(v) &= d_w \frac{v^2}{v^2+\nu}, & R^w &= a_w. \end{aligned} \tag{S10}$$

Results are shown in Fig. S1a. Parameters in Eqs. (S10) are fixed at $a_u = 2.5, b_u = 3, c_u = 1.2, a_v = 0.2, c_v = 1.2, d_v = 1, a_w = 0.75, d_w = 1$ and $\mu = \nu = 0.125$. A uniform steady state is found at $(u_0, v_0, w_0) \simeq (0.509, 0.612, 0.497)$. The oscillatory Turing instability is observed as σ_u is increased.

Model E. The food chain (Fig. 1a) with the Holling type II dependence for prey and a linear function for predator:

$$\begin{aligned} Q^u(u) &= a_u - b_u u, & R^u(u,v) &= c_u \frac{v}{u+\mu}, \\ Q^v(u,v) &= C_v + a_v \left(1 - c_v \frac{v}{u}\right), & R^v(v,w) &= C_v + d_v \frac{w}{v+\nu}, \\ Q^w(v,w) &= C_w + a_w \left(1 - d_w \frac{w}{v}\right), & R^w &= C_w. \end{aligned} \tag{S11}$$

Results are shown in Fig. S1b. Parameters in Eqs. (S11) are fixed at $a_u = 3, b_u = 1, c_u = 1, a_v = 6, c_v = 1/6, d_v = 1, a_w = 4, d_w = 0.25$ and $\mu = \nu = 0.25$. A uniform steady state is found at $(u_0, v_0, w_0) \simeq (1.084, 2.557, 10.23)$. The oscillatory Turing instability takes place in this system as σ_w is increased.

Model F. The food chain (Fig. 1a) with the Holling type III dependence for prey and a linear function for predator:

$$\begin{aligned} Q^u(u) &= a_u - b_u u, & R^u(u,v) &= c_u \frac{uv}{u^2+\mu}, \\ Q^v(u,v) &= C_v + a_v \left(1 - c_v \frac{v}{u}\right), & R^v(v,w) &= C_v + d_v \frac{vw}{v^2+\nu}, \\ Q^w(v,w) &= C_w + a_w \left(1 - d_w \frac{w}{v}\right), & R^w &= C_w. \end{aligned} \tag{S12}$$

Results are shown in Fig. S1c. Parameters in Eqs. (S12) are fixed at $a_u = 3, b_u = 1, c_u = 1.5, a_v = 8, c_v = 0.25, d_v = 2.5, a_w = 5, d_w = 0.4$ and $\mu = \nu = 0.25$. A uniform steady state is $(u_0, v_0, w_0) \simeq (1.477, 1.672, 4.179)$. The oscillatory Turing instability is found as σ_w is increased.

Note that the constants $C_{v,w}$ enter both into the reproduction and death rates of species V and W in Eqs. (S11) and (S12). Therefore, they become cancelled in the expressions for G and H. Their numerical values are not relevant and are not provided.

Model A with one immobile species. As implied by the sufficient conditions (S2)-(S4), the oscillatory Turing instability is possible even if one of the species is immobile. Figure S2 shows results for the case that the intermediate species V is immobile, i.e. $\sigma_v = 0$ (Fig. S2a) and that the top predator W is immobile, i.e. $\sigma_w = 0$ (Fig. S2b). Parameters are the same as those used in the main text. The oscillatory Turing instability is observed as σ_u is increased in both cases.

Thus, the oscillatory Turing instability could be observed for different food web architectures (Figs. 2 and 3 in the main text) and for different nonlinearities in the reproduction and death rates (Fig. S1). Moreover, the oscillatory Turing patterns were observed even when one of the three species was immobile (Fig. S2). In all considered systems, oscillatory Turing bifurcations were supercritical.

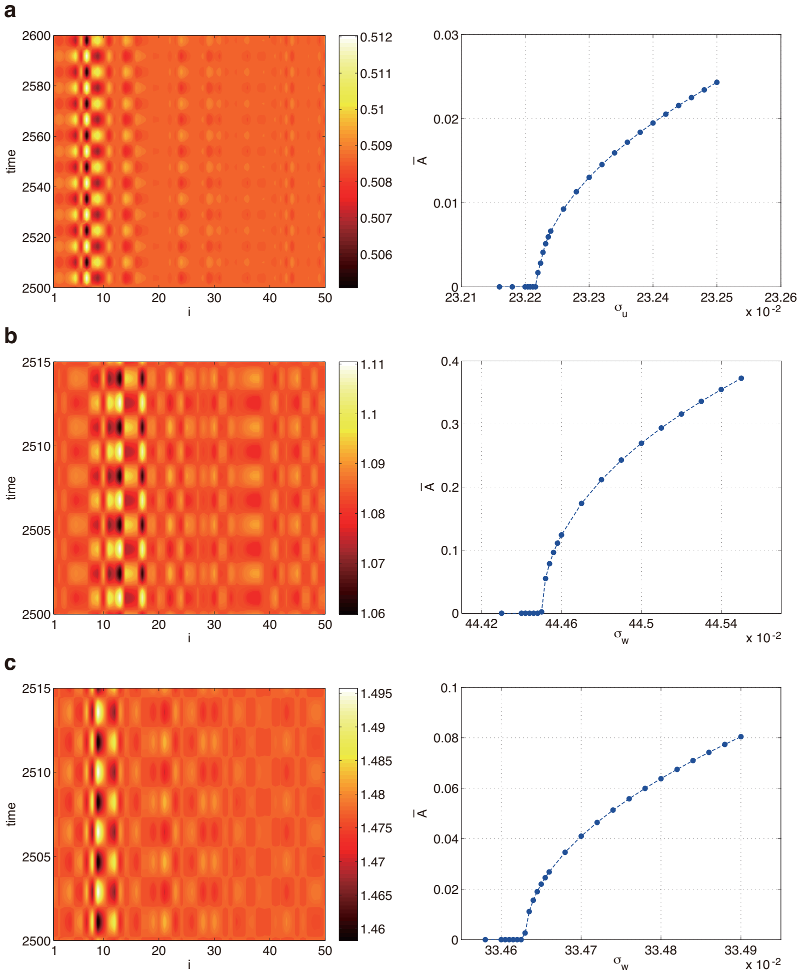

FIG. S1: Oscillatory Turing instabilities in (a) Model D, (b) Model E and (c) Model F. Left panels show final oscillatory Turing patterns. Right panels show the amplitude $\bar{A}$ as a function of σ_u or σ_w. Dispersal mobilities are fixed at (a) $\sigma_v = \sigma_w = 0.01$ and $\epsilon = 0.34$. (b) $\sigma_u = \sigma_v = 0.01$ and $\epsilon = 1.6$. (c) $\sigma_u = \sigma_v = 0.01$ and $\epsilon = 1.6$. For the final patterns, (a) $\sigma_u = 0.2323$, (b) $\sigma_w = 0.4446$ and (c) $\sigma_w = 0.3347$ are used.

III. NUMERICAL SIMULATIONS OF CHEMICAL REACTION-DIFFUSION NETWORKS

In chemical systems, autocatalytic reactions and diffusion may induce the Turing instability. The chemical Brusselator [4] and the Oregonator [5] are typical mathematical models to describe autocatalytic reactions and their numerical investigations are extensively used for exploring pattern formations in chemical systems. The Brusselator was originaly proposed to examine chemical oscillatory dynamics. It is a hypothetical model, which gives a rich variety of chemical dissipative structures. In contrast to the Brusselator, the Oregonator has been proposed to describe the real Belousov-Zhabotinsky reaction.

Both chemical models have been previously extended to explore the oscillatory Turing instability (wave instability) in continuous media [6, 7]. It was suggested that a chemical reaction in which the activator is reversibly transformed into an unreactive chemical species can explain wave patterns observed in the BZ aerosol OT system [6]. Therefore, an additional third component taking such reversible transformation was added to the Brusselator. For the Oregonator, which originally contains three components, an additional reversal reaction was added. Thus, extended Brusselator and Oregonator were constructed [7].

Here, we consider the behaviors of such extended models in network-organized systems. The extended network

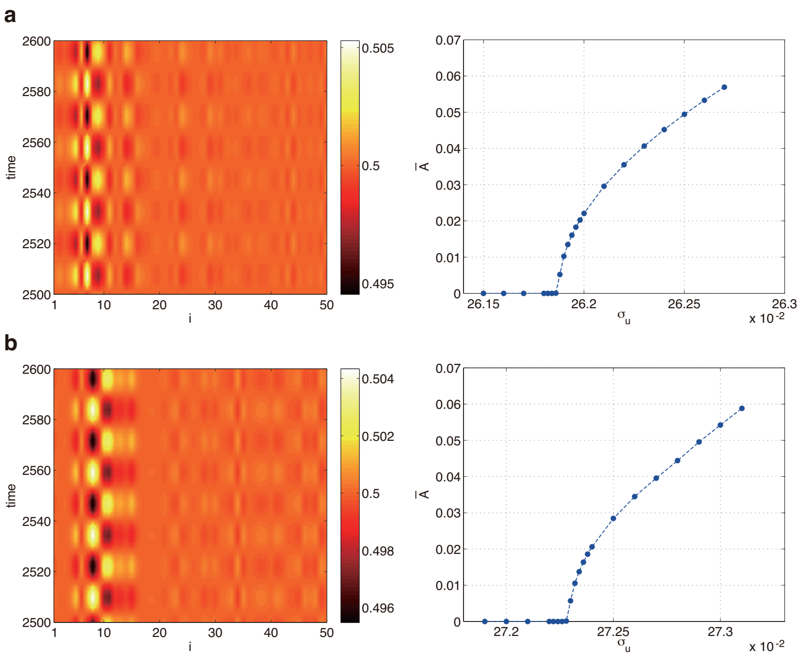

FIG. S2: Oscillatory Turing instabilities in food chains (Model A) model with only two mobile species. Left panels show final oscillatory Turing patterns. Right panels show the amplitude $\bar{A}$ as a function of σ_u. (a) Intermediate predator V is immobile ($\sigma_v = 0, \sigma_w = 0.01$). (b) Top predator W is immobile ($\sigma_v = 0.01, \sigma_w = 0$). Oscillatory Turing patterns in left panels are observed at (a) $\sigma_u = 0.262$ (b) $\sigma_u = 0.2724$ with the overall dispersal mobility $\epsilon = 0.4$.

Brusselator model is described by equations

$$\begin{cases} \dfrac{du_i}{dt} = a - (1+b)u_i + {u_i}^2 v_i - cu_i + dw_i + \epsilon\sigma_u \displaystyle\sum_{j=1}^{N} L_{ij} u_j, \\ \dfrac{dv_i}{dt} = bu_i - {u_i}^2 v_i + \epsilon\sigma_v \displaystyle\sum_{j=1}^{N} L_{ij} v_j, \\ \dfrac{dw_i}{dt} = cu_i - dw_i + \epsilon\sigma_w \displaystyle\sum_{j=1}^{N} L_{ij} w_j. \end{cases} \tag{S13}$$

We fix parameters as $a = 1, b = 2.9, c = 1$ and $d = 1$, yielding a steady state $(u_0, v_0, w_0) = (1, 2.9, 1)$. The extended network Oregonator model is given by

$$\begin{cases} \dfrac{du_i}{dt} = \dfrac{1}{\epsilon_0}\left[u_i - {u_i}^2 - pv_i \dfrac{u_i - q}{u_i + q} - cu_i + dw_i\right] + \epsilon\sigma_u \displaystyle\sum_{j=1}^{N} L_{ij} u_j, \\ \dfrac{dv_i}{dt} = u_i - v_i + \epsilon\sigma_v \displaystyle\sum_{j=1}^{N} L_{ij} v_j, \\ \dfrac{dw_i}{dt} = \dfrac{1}{\epsilon_1}[cu_i - dw_i] + \epsilon\sigma_w \displaystyle\sum_{j=1}^{N} L_{ij} w_j, \end{cases} \tag{S14}$$

with parameters $p = 0.95, q = 0.01, c = 0.2, d = 1, \epsilon_0 = 0.35$ and $\epsilon_1 = 2$, yielding a steady state $(u_0, v_0, w_0) \simeq (0.161, 0.161, 0.032)$.

Numerical results are shown in Fig. S3. In both systems, the oscillatory Turing instability takes place when increasing the diffusion mobility σ_w of reactant w. The bifurcation is supercritical and leads to localized oscillations in chemical networks.

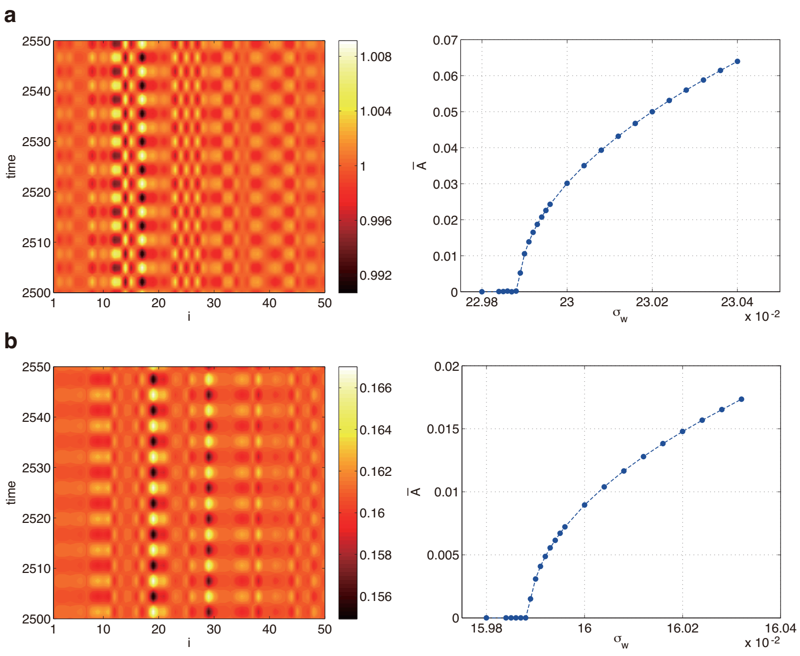

FIG. S3: Oscillatory Turing instability in (a) the extended Brusselator model and (b) the extended Oregonator model. Left panels show final oscillatory Turing patterns. Right panels show the amplitude $\bar{A}$ as a function of σ_w. Diffusional mobilities are fixed at (a) $\epsilon = 0.35, \sigma_u = 0.01$ and $\sigma_v = 0.01$, (b) $\epsilon = 0.5, \sigma_u = 0.01$ and $\sigma_v = 0.01$. For the final patterns, (a) $\sigma_w = 0.23$ and (b) $\sigma_w = 0.16$ are used.

---

[1] Hata, S., Nakao, H. & Mikhailov, A. S. Sufficient conditions for wave instability in three-component reaction-diffusion systems. accepted in *Progr. Theor. Exp. Phys.* (2013).

[2] Holling, C. S. Some characteristics of simple types of predation and parasitism. *The Canadian Entomologist* **91**, 385-398 (1959).

[3] Murray, J. D. *Mathematical Biology* (Springer, 2003).

[4] Glandsdorff, P. & Prigogine, I. *Thermodynamic Theory of Structure, Stability and Fluctuations* (Wiley, 1971).

[5] Field, R. J. & Noyes, R. M. Oscillations in chemical systems. IV. Limit cycle behavior in a model of a real chemical reaction. *Journal of Chemical Physics* **60**, 1877-1884 (1974).

[6] Vanag, V. K., & Epstein, I. R. Pattern formation in a tunable medium: The Belousov-Zhabotinsky reaction in an aerosol OT microemulsion. *Phys. Rev. Lett.* **87**, 228301 (2001).

[7] Yang, L., Dolnik, M., Zhabotinsky, A. M. & Epstein, I. R. Pattern formation arising from interactions between Turing and wave instabilities. *Journal of Chemical Physics* **117**, 7259 (2002).
